# 糖酵解与肺癌相关研究进展

**DOI:** 10.3779/j.issn.1009-3419.2012.04.07

**Published:** 2012-04-20

**Authors:** 博宁 刘, 清华 周

**Affiliations:** 300052 天津，天津医科大学总医院，天津市肺癌研究所，天津市肺癌转移与肿瘤微环境重点实验室 Tianjin Key Laboratory of Lung Cancer Metastasis and Tumor Microenvironment, Tianjin Lung Cancer Institute, Tianjin Medical University General Hospital, Tianjin 300052, China

肺癌是全球发病率和病死率最高的恶性肿瘤。男性中肺癌占新发肿瘤病例的17%以上，约占癌症死亡总数的23%，且发病率呈上升趋势^[1]^。肺癌患者中约80%为非小细胞肺癌(non-small cell lung cancer, NSCLC)。NSCLC预后较差，主要与局部复发及远处转移有关，而较多患者就诊时已经发生局部侵润(37%)和远处转移(38%)^[2]^。在NSCLC等实体肿瘤的生长过程中，癌细胞增殖迅速造成癌组织微环境始终处于相对低氧状态。此现象在实体肿瘤中普遍发生，是肿瘤微环境最重要的特征之一，其主要机制与肿瘤生长不一致造成肿瘤血管结构功能的异常有关。低氧可导致肿瘤细胞的基因及蛋白质表达发生改变，致使肿瘤生长受限，发生细胞周期停滞、凋亡和坏死；同时，低氧也可通过诱导新生血管、加强糖酵解、激活生长因子而抑制凋亡^[3]^。近期研究表明，低氧下糖酵解的增强与NSCLC等实体肿瘤的发展、侵袭和转移密切相关。本文就有氧糖酵解中的重要调控因子缺氧诱导因子1(hypoxia-inducible factor-1, HIF-1)和葡萄糖转运体(glucose transporter, GLUT)与肺癌的相关性进行综述，为肺癌防治提供新的思路和理论基础。

## 1 瓦博格效应与肿瘤

细胞的能量获取主要源于糖代谢。葡萄糖在体内氧化分解的途径包括氧化磷酸化和糖酵解。正常细胞在有氧条件下主要通过氧化磷酸化产生ATP，而在缺氧条件下则主要通过糖酵解产生ATP。1924年首次提出瓦博格效应(warburg effect)，指出细胞在癌变时主要通过糖酵解获取ATP，即在氧充足的条件下其能量代谢的主要途径由三羧酸循环切换为有氧糖酵解^[4]^。有氧糖酵解是肿瘤恶变过程中最基础的代谢改变，此现象广泛存在于各种肿瘤组织中，它能够促进肿瘤细胞的增殖、增强侵袭性并参与介导耐药^[4-6]^。

大量研究^[7, 8]^证实瓦博格效应的产生及恶性细胞对其依赖程度由细胞内部和外部环境共同决定，并由多基因(如癌基因*RAS*、*C-MYC*、*AKT*、*BCR*/*ABL*和抑癌基因*p53*等)及信号激酶(如AMP激酶、AKT激酶等)共同调控。其中许多癌基因和抑癌基因自发性改变参与此能量代谢调控过程^[9, 10]^。此外，参与有氧糖酵解调控的关键因子还有HIF-1、GLUT1和GLUT3、乳酸、糖酵解酶类、碳酸酐酶等，其中尤以HIF-1和GLUT1的过度表达与NSCLC的增殖和转移密切相关^[11-14]^。

## 2 HIF-1与NSCLC

### 2.1 HIF-1活性的调节

缺氧可分为急性缺氧(灌注限制性缺氧)和慢性缺氧(弥散限制性缺氧)^[15]^，可引发肿瘤细胞基因及蛋白质表达发生改变，诱导血管新生、加强糖酵解、激活生长因子、抑制凋亡，使肿瘤细胞克服并适应低氧和营养缺乏的微环境。HIF-1是一种介导低氧适应性反应的转录因子，可激活许多低氧反应性基因的表达，是在低氧条件下维持氧稳态的关键性物质。HIF-1由HIF-1α和HIF-1β两个亚单位构成，HIF-1α为主要的氧调节亚基，低氧主要通过调节其蛋白水平来影响HIF-1的转录调节作用；HIF-1β则对氧的依赖性较弱，其碱基可以特异性识别靶基因上DNA的结合位点，发挥转录因子的激活特性。HIF-1作为缺氧相对特异性的内源性标记物和缺氧诱导过程中最重要的转录因子，其表达程度可代表肿瘤瞬时缺氧的状况。

近期研究表明，在多种人类肿瘤中癌基因可不依赖缺氧诱导，直接活化HIF-1和其它葡萄糖代谢通路元件。Gatenby等^[16]^发现胰岛素样生长因子(insulin-like growth factor, IGF)的刺激以及V-SRC、C-SRC和RAS的激活均可上调HIF-1的表达；此外HER2通路或PI3-AKT通路的激活也可促进HIF-1的合成^[7]^。PI3Ka或AKT1的活性突变所致的哺乳动物雷帕霉素靶蛋白(mammalian target of rapamycin, mTOR)信号通路活化，可以促进HIF-1的转录和翻译^[17]^。除了癌基因突变外抑癌基因失活也参与了细胞能量代谢调节，如*PTEN*、*LKB1*、*PML*和*TSC1*/*TSC2*等抑癌基因的任一功能性失活都可以通过mTOR信号通路促进HIF-1的转录和翻译，诱导代谢相关的基因表达^[18, 19]^。

### 2.2 HIF-1与细胞代谢相关性

HIF-1所调控的基因涉及细胞能量代谢、离子代谢、肿瘤的侵袭和转移、干细胞的稳态维持、儿茶酚胺代谢及血管的发生等诸多方面^[20, 21]^。有研究^[22]^表明低氧反应中有55个相关基因上调，其中89%受HIF-1调控；119个相关基因下调，其中17%受HIF-1调控；其中所有的糖酵解酶类均被HIF-1上调。低氧反应中HIF-1介导的关键因子包括血管内皮生长因子(vascular endothelial growth factor, VEGF)、GLUT1和GLUT3、糖酵解酶类、碳酸酐酶、乳酸脱氢酶A、单羧基转运体4、丙酮酸脱氢酶激酶1等，涉及糖酵解、有氧呼吸、线粒体自噬等多方面细胞功能^[16, 18, 22]^。这些缺氧诱导的基因启动子上都含有低氧反应元件(hypoxic response elements, HRE)。HRE是一个增强子，在缺氧条件下细胞核产生的HIF-1与靶基因*HRE*中的HIF-1结合位点结合促进转录，从而引起细胞对缺氧产生一系列的反应^[23]^。众所周知，AKT可促进肿瘤细胞代谢改变、增强恶性程度，其调控的代谢因子包括与线粒体及凋亡抵抗相关的己糖激酶(hexokinase, HK)、糖酵解限速环节之一的6-磷酸果糖激酶1(6-phosphofructokinase 1, PFK1)以及分布最为广泛的葡萄糖转运体GLUT1^[24]^。HIF-1则可上调GLUT1和GLUT3的表达，激活HK1和HK2的转录，上调HK的合成^[25]^。

HIF-1可调节能够将丙酮酸转化为乳酸的乳酸脱氢酶A和将乳酸转运出细胞的单羧酸转运蛋白的表达^[25, 26]^。还有研究^[27]^发现HIF-1也参与丙酮酸脱氢酶激酶表达的调控。丙酮酸脱氢酶激酶通过催化丙酮酸脱氢酶磷酸化使之失去活性，因此丙酮酸脱氢酶激酶的激活可以阻止丙酮酸进入线粒体，减少参与三羧酸循环的丙酮酸量，从而减少NADH和FADH2电子链的传递。这是细胞对低氧适应的主要机制之一。肿瘤细胞中HIF-1通过上调糖转运和糖酵解酶的相关基因，促使肿瘤在低氧时采用糖酵解途径^[28]^。而相关编码蛋白和肿瘤抑制基因突变造成的HIF-1结构性表达，可使肿瘤在氧充足时仍然采用糖酵解的方式进行能量代谢^[29]^。

### 2.3 HIF-1与NSCLC的相关性研究

HIF-1在肿瘤的发生和转移中扮演着重要角色，其基因多态性与NSCLC预后密切相关^[12]^。Zhong等^[30]^应用免疫组化方法检测了HIF-1在多种肿瘤中的表达，发现90%的NSCLC组织中HIF-1表达升高，而癌旁组织中未见HIF-1表达。Li等^[31]^应用免疫组化方法检测CCR7、HIF-1和HIF-2在94例NSCLC组织中的表达，发现CCR7、HIF-1和HIF-2的阳性表达与NSCLC临床分期和淋巴结转移相关；体外实验发现缺氧可以通过诱导BE1和A549肺癌细胞的HIF-1和HIF-2表达而上调CCR7的表达；应用siRNA技术抑制A549肺癌细胞HIF-1和HIF-2表达则可以下调CCR7表达，并抑制细胞的侵袭转移潜能。Zuo等^[32]^应用免疫组化SP法检测HIF-1和VEGF-C蛋白在48例NSCLC癌组织及癌旁组织的表达，结果发现HIF-1和VEGF-C蛋白在癌组织的阳性表达率分别为70.8%(34/48)和68.8%(33/48)，明显高于癌旁组织(12.5%和16.7%，*P* < 0.05)；HIF-1和VEGF-C蛋白在癌组织的阳性率与TNM分期及淋巴结转移相关。Wu等^[33]^应用组织化学和免疫组织化学方法检测160例NSCLC组织和20例正常肺组织标本的血管生成拟态以及CD82/KAI1、HIF-1蛋白的表达，发现NSCLC组织中CD82/KAI1、HIF-1蛋白及血管生成拟态阳性率分别为37.5%、48.8%和36.9%，而正常肺组织分别为95.0%、0和0；HIF-1蛋白及血管生成拟态之间具有相关性，血管生成拟态与HIF-1蛋白过表达与NSCLC不良预后相关。这些研究结果提示HIF-1在NSCLC的侵袭迁移过程中扮演着重要角色，HIF-1提高NSCLC侵袭迁移潜能的机理可能与其促进NSCLC细胞有氧糖酵解相关。

### 2.4 HIF-1在肿瘤治疗中的应用

HIF-1抑制剂类药物总体可分为小分子HIF-1活性抑制剂及相关信号通路阻断剂两类。较早发现的小分子HIF-1活性抑制剂有可溶性鸟苷酸环化酶抑制剂YC-1、热休克蛋白90抑制剂格尔德霉素、苯甲酸类似物以及缺氧性细胞毒素TX-402等。近期研究^[34]^还发现异硫氰酸苯乙酯(phenethyl isothiocyanate, PEITC)能通过减少HIF-1的RNA翻译，有效抑制HIF-1蛋白积聚，诱导其内源靶基因*CAIX*、*GLUT1*、*BNIP3*及*VEGF*发生改变。在乳腺癌中使用HIF抑制剂地高辛和吖啶黄，可有效抑制低氧中赖氨酰氧化酶(lysyl oxidase, LOX)和赖氨酰氧化酶样(lysyl oxidase-like, LOXL)的蛋白表达上调，阻碍胶原蛋白交联，减少骨髓源性干细胞聚集，从而干扰转移微环境形成，抑制乳腺癌细胞的肺转移^[35]^。还有研究^[36, 37]^证实脯氨酰羟化酶(prolyl hydroxylase, PHD)是催化HIF-1降解的限速酶，使用PHD2的强效激活剂KRH102053及其结构类似物KRH102140可降解HIF-1蛋白，抑制其相关调控基因如*VEGF*、醛缩酶A、烯醇酶1和单羧酸转运蛋白4等，降低多种肿瘤细胞的迁移侵袭活力。在肺癌方面近几年也出现了大量以HIF-1为靶点的研究。多种NSCLC中发现拓扑异构酶Ⅰ抑制剂拓扑替康(topotecan)及拓扑异构酶Ⅱ抑制剂依托泊苷(etoposide)能够有效抑制HIF-1蛋白表达及AKT磷酸化，且呈现时间周期依赖性^[38]^。在A549细胞中穿心莲内酯可通过TGF1通路上调PHD2，使HIF-1发生快速泛素依赖性降解，下调VEGF的蛋白表达，抑制肿瘤微血管生成^[39]^。PX-478作为HIF-1的强效小分子抑制剂，被证实在体内及体外模型中均可抑制肺腺癌及小细胞肺癌的细胞活力，被建议纳入肺癌患者Ⅰ期临床试验^[40]^。还有研究^[41]^发现，雷帕霉素能够通过促进HIF-1降解来抑制生存蛋白(survivin)的表达，诱导多种NSCLC细胞系发生凋亡。

基因治疗方面已有多项研究证实利用RNA干扰等相关技术特异性抑制HIF-1活性，可有效抑制多种恶性肿瘤细胞活性。Kamlah等^[42]^研究发现，静脉注射HIF-1-siRNA和HIF-2-siRNA可以有效延长Lewis肺癌荷瘤裸鼠的生存期，抑制肿瘤生长并减少血管生成。在宫颈癌细胞中靶向沉默HIF-1可明显下调*GLUT1*和*HK2*的基因表达水平，降低糖酵解活性并促进细胞凋亡，抑制体内肿瘤生长^[43]^。此外，一些HIF-1上游调控因子也在基因治疗方面受到关注。研究^[44]^表明酪蛋白激酶2(casein kinase 2, CK2)siRNA可通过激活p53降低缺氧环境中HIF-1的活性；靶向激活p53则能有效抑制糖酵解中C-MYC、HIF-1和GLUT的表达，降低能量代谢水平，从而特异性杀伤癌细胞^[45]^。

## 3 GLUT1与肺癌

### 3.1 GLUT1与肿瘤的相关性

葡萄糖转运体蛋白在哺乳动物细胞糖代谢过程中扮演着重要的角色。葡萄糖是水溶性物质，需要借助GLUT转运通过细胞磷脂双分子层进入胞浆，这是葡萄糖代谢过程中第一个限速步骤。目前发现GLUT家族成员有14种，其中GLUT1、GLUT3和GLUT4与葡萄糖有较高的亲和力，在正常生理条件下可高效地转运葡萄糖。GLUT1在体内分布最广，各组织中均存在表达^[46]^。在缺血、缺氧等情况下可出现组织中GLUT1异常增高。由于在恶性肿瘤细胞中常常检测到GLUT1和GLUT3的过表达，所以有学者提出可以将GLUTl作为内源性缺氧标志物来检测肿瘤内的缺氧情况^[47]^。近年来发现，GLUT1过度表达与多种肿瘤如肾癌^[48]^、乳腺癌^[49]^、直肠癌^[50]^有关，且与NSCLC的关系最为密切，与其肿瘤组织类型、分化程度、肿瘤体积、淋巴结转移及患者预后均相关。

GLUT在肿瘤中的表达及功能受许多因子调控，其中癌基因和肿瘤缺氧作用最强，GLUT1的广泛过表达可能是这两个因素相互作用的结果。现已知，GLUT1的表达受低氧环境和HRE/HIF复合物双重调控，代谢抑制使GLUT1转化为单向转运，确保低氧诱导葡萄糖摄取增加。在癌细胞中抑制氧化磷酸化的协同作用可上调GLUT1，与此同时HIF-1也可诱导其高表达。Williams等^[51]^研究发现在HIF-1缺失的中国仓鼠卵巢癌异种接种物细胞和HIF-1缺失的鼠肝细胞瘤细胞中GLUT1不表达，而野生型移植物坏死区域周围的细胞GLUT1呈过表达。在结构性表达HIF-1的肾脏透明细胞癌中，GLUT1和HIF-1调控的基因表达上调，这提示HIF-1对GLUT1的最终表达水平有决定性作用。

### 3.2 GLUT1与NSCLC的相关性

研究^[14]^表明卵巢癌和NSCLC细胞中缺氧可诱导脱氧葡萄糖(fluorodeoxyglucose, FDG)摄入量增加及GLUT1蛋白表达上调。Ai等^[11]^应用免疫组织化学方法检测84例NSCLC组织中GLUT1和HIF-1蛋白的表达，发现GLUT1和HIF-1的阳性表达率分别为95.2%(80/84)和96.4%(81/84)，且具有相关性。这表明GLUT1可能与NSCLC葡萄糖的摄取及糖酵解有关，HIF-1可能参与了GLUT1的上调。Younes等^[52]^应用抗GLUT1和抗GLUT3多克隆抗体对289例Ⅰ期NSCLC组织进行免疫组织化学检测，结果显示GLUT1阳性率为83%(239/289)，GLUT3阳性率为21%(61/289)，GLUT1和GLUT3过表达与Ⅰ期NSCLC较差生存期相关，GLUT1阳性可作为NSCLC恶性侵袭的标记物。Sasaki等^[53]^发现GLUT1在NSCLC组织中的表达与*KRAS*基因突变有关，GLUT1蛋白的过表达与NSCLC的侵袭表型相关，GLUT1阳性的NSCLC患者预后明显差于GLUT1正常表达者。Kaira等^[54]^对147例原发性NSCLC组织进行免疫组织化学检测，发现GLUT1和HIF-1在未分化原发性NSCLC中的表达明显高于高分化组和低分化组。上述研究表明GLUT1的表达与NSCLC细胞的恶性增殖和侵袭转移性能密切相关，侵袭性强、生长旺盛的NSCLC细胞常伴有葡萄糖代谢增加。

### 3.3 GLUT1在肿瘤诊断及治疗中的应用

如今GLUT1已被普遍认为是细胞恶性病变的早期标志，可用作早期癌前病变的诊断依据。大量研究^[55]^表明GLUT1的高表达与癌前病变发展无相关性，但与肿瘤进展、分化以及患者预后及生存率存在一定关联。在相关肿瘤治疗方面，研究普遍认为利用GLUT1肿瘤高表达的特点，一方面可应用化疗药物结合葡萄糖，通过GLUT1高效转运来提高药物摄取效率，达到对肿瘤细胞的有效杀伤；另一方面也可通过直接阻断GLUT1在肿瘤细胞中的表达，降低细胞的能量代谢效率而发挥抑癌功效。Subbarayan等^[56]^发现，一氧化氮可通过与氧基团结合形成亚硝基蛋白，损伤DNA，而目前获得的亚硝基乙酰青霉胺(S-nitroso-N-acetyl-penicillamine, SNAP)并不能直接靶向肿瘤细胞，但如将其与葡萄糖结合形成2-gluSNAP复合物，则能大大提高其杀伤肿瘤细胞的能力。在GLUT1相关分子抑制剂探索方面，Wardell等^[57]^发现使用组蛋白脱乙酰基酶抑制剂(histone deacetylase inhibitors, HDACIs)可靶向抑制骨髓瘤细胞中GLUT1介导的葡萄糖转运，通过改变碳源摄取途径建立不利于肿瘤细胞快速生长存活的能量代谢模式提高疗效。Cao等^[58]^研究表明，联合GLUT抑制剂根皮素和化疗药物柔毛霉素可有效提高癌细胞的化疗敏感度，降低抗药性。Lemoine等^[59]^应用泛脱乙酰基酶(pan-deacetylase, DAC)抑制剂LBH589作用霍杰金淋巴瘤细胞系，发现其能够通过下调HIF-1及下游靶基因*GLUT1*与*VEGF*的表达以有效杀伤癌细胞，有望将LBH589联合mTOR抑制剂治疗此类肿瘤患者；同时LBH589也可参与矫正GLUT1相关的FDG-PET临床影像诊断。

在基因治疗方面较多研究选择以GLUT1上游转录因子作为靶点进行干预。有研究^[60]^表明应用siRNA技术沉默U251、U87和U373神经胶质瘤细胞的HIF-1后，GLUT1和VEGF蛋白表达水平明显下降；相应裸鼠体内实验中siRNA治疗组比对照组的肿瘤体积减小了约50%。

## 4 问题与展望

肿瘤生长不一致可导致肿瘤血管功能和结构异常，致使肿瘤组织中出现区域性急性缺氧和慢性缺氧，引起肿瘤细胞的基因及蛋白质表达发生改变，发生细胞生长受限和死亡；同时，低氧也可诱导新生血管、增强糖酵解、抑制凋亡、激活生长因子。葡萄糖的摄取和糖酵解的增强造成肿瘤细胞恶性转化、侵袭转移和对放化疗等治疗的抵抗，使目前的治疗方法难以达到理想的效果，为肿瘤的治疗带来了难题，也提供了新的切入点。目前FDG-PET显像、氧电极应用和肿瘤内源性缺氧标志物的检测已经为肿瘤的早期诊断、治疗前后效果的评估和预后的判断提供了新的方法。然而，尽管有氧糖酵解是肿瘤最明显的能量代谢特征，仍存在一部分肿瘤细胞无法用FDG-PET成像即“PET阴性”肿瘤。是否此类肿瘤细胞并非依赖糖代谢，而是依靠其它形式的生物能量还有待探索。目前HIF-1和GLUT1对糖酵解的调控机制尚不完全清楚，但随着对HIF-1和GLUT1与NSCLC相关研究的深入，HIF-1和GLUT1有望成为NSCLC治疗的新靶点。
